# Translation and Validation of the Sinhalese Version of the Brief Medication Questionnaire in Patients with Diabetes Mellitus

**DOI:** 10.1155/2018/7519462

**Published:** 2018-05-23

**Authors:** P. Ranasinghe, R. Jayawardena, P. Katulanda, G. R. Constantine, V. Ramanayake, P. Galappatthy

**Affiliations:** ^1^Department of Pharmacology, Faculty of Medicine, University of Colombo, Colombo, Sri Lanka; ^2^Department of Physiology, Faculty of Medicine, University of Colombo, Colombo, Sri Lanka; ^3^Department of Clinical Medicine, Faculty of Medicine, University of Colombo, Colombo, Sri Lanka

## Abstract

**Background:**

Adherence to long-term therapy for diabetes remains low. Accurately measuring adherence is the primary step in improving adherence. We translated and validated the Sinhalese version of the Brief Medication Questionnaire (BMQ) in patients with diabetes.

**Methods:**

The study was conducted at the National Hospital of Sri Lanka between April and December 2017, including 165 patients with diabetes. BMQ was translated into Sinhalese using the translation-back translation method. The translated questionnaire validation included evaluation of internal consistency, temporal stability, and performance in regard to a gold standard (HbA1c).

**Results:**

Mean age (±SD) was 60.6 ± 11.1 years, and 46.1% were males. Mean duration of diabetes in the participants was 13.4 ± 7.8 years. Mean HbA1c was 8.3 ± 1.7%, with poor glycaemic control (HbA1c ≥ 8.5%) identified in 41.8%. Medication adherence measured by the BMQ regimen, belief, and recall screens were 39.4%, 75.8%, and 18.8%, respectively. In the analysis of temporal stability, the overall BMQ and the regimen, belief, and recall screens demonstrated good concordance between test and retest with significant gamma correlation coefficients of *r* = 0.85 (*p* < 0.001), *r* = 0.81 (*p* < 0.001), *r* = 0.84 (*p* < 0.001), and *r* = 0.91 (*p* < 0.001), respectively. The overall BMQ had a Cronbach *α* coefficient of 0.65 (95% CI: 0.61–0.70). The questionnaire performance with regards to the gold standards for the overall BMQ AUC was 0.73 (95% CI 0.65–0.80), while the BMQ regimen screen AUC was 0.61 (95% CI 0.53–0.70). The overall BMQ score with a cutoff value of 2 presented better equilibrium between sensitivity and specificity for the gold standard. Those with low adherence had a significantly higher percentage of poor glycaemic control (HbA1c ≥ 8.5%).

**Conclusion:**

The translated questionnaire demonstrated good reliability (internal consistency), temporal stability (test-retest reliability), and validity when assessed using a gold standard for disease control. Using culturally validated tools to evaluate adherence may help clinicians to identify low adherence and institute corrective measures.

## 1. Introduction

It is estimated that over 415 million people have diabetes around the world, with nearly 78 million people living in the Southeast Asian region, a figure which is expected to rise to 140 million by 2040 [1]. Diabetes is also increasingly becoming prevalent in Sri Lanka. The International Diabetes Federation (IDF) estimates that nearly 10.7% of the adult population in Sri Lanka are suffering from diabetes, with an estimated 4 million adults with dysglycaemia [2]. The World Health Organization (WHO) predicts that diabetes will become the 7th leading cause of death in the world by the year 2030 [3]. Poor glycaemic control has been directly linked to the macro-/microvascular complications and premature mortality due to diabetes, with a 21% increase in the total morality for each 1% increase in the HbA1c [4]. Similar to most chronic noncommunicable diseases, adequate management of diabetes is also dependent on proper medication adherence. Adherence to long-term therapy for chronic illnesses in developed countries averages 50% and is estimated to be even lower in developing countries [5].

It is very important to encourage medication adherence for a better outcome of the disease. One of the primary steps in improving medication adherence is reliably and accurately measuring adherence [6]. Several methods are available for the assessment of adherence; however, accurate measurement continues to be difficult and each available method has its own advantages and disadvantages [7]. Medication adherence assessment methods are categorized as direct and indirect, with direct methods including measurement of the level of the target drug or metabolite in the blood, measurement of a biological marker in the blood, and directly observed therapy [7]. Most commonly used indirect methods include patient self-reports via a validated questionnaire, pill counts, and pharmacy refills [7]. Although direct methods are considered to be more robust than indirect methods, they also have limitations, including the practical application in clinical settings.

Self-reported instruments are considered to be convenient, inexpensive, easy to administer, and effective. One of the most widely used and accepted patient self-reported instruments is the Brief Medication Questionnaire (BMQ) [8]. It consists of three different screens, a 5-item regimen screen, a 2-item belief screen, and a 2-item recall screen. These screens assess how patients took each of their medications in the past week, on drug efficacy and bothersome features and remembering difficulties, respectively [8]. Svarstad et al. further reviewed that, with its ability to allow self-administration, evaluate multidrug regimens, and reduce practitioner's training, this questionnaire is popular among healthcare professional [9]. The original English version of BMQ has been validated in patients with hypertension [9]. The BMQ has also been validated for use in several countries and has been found to be a valid and reliable scale to measure adherence in patients with diabetes, epilepsy, and myocardial infarction [7]. It has been translated to Tamil [10]. Sinhala is the language spoken by majority of the Sri Lankan population (>70%) [11]. However, a validated Sinhala version of the BMQ is not currently available for use in patients with diabetes. Hence, the present study aims at translating and validating the Sinhalese version of the BMQ in patients with diabetes.

## 2. Methods

### 2.1. Study Population and Sampling

The study was conducted at the medical clinic of the University Medical Unit at the National Hospital of Sri Lanka (NHSL), Colombo, Sri Lanka between April and December 2017. The study was approved by the Ethics Review Committee of the Faculty of Medicine, University of Colombo, Sri Lanka and was conducted in compliance with the Declaration of Helsinki. Based on evidence from previous research, a subject-item ratio of 15 was used to calculate the sample size [12]. Since the BMQ contains 10 questions, the required sample size was estimated to be 150 (10 items^∗^15 subjects). We estimated a dropout ratio of 10% based on our experience from previous similar research projects. Hence, the final sample size was calculated as 165 patients with diabetes satisfying the inclusion/exclusion criteria given below. A systematic random-sampling method of eligible patients was used to select participants until the required sample size was achieved. The study team visited the medical clinics of the University Medical Unit, twice per week. On each day of the visit, we obtained a list of the patients with diabetes from the clinic register. From this list, the first patient was selected randomly, and thereafter every 3rd patient was selected for the study and recruited after confirming eligibility. Informed written consent was obtained from all participants prior to recruitment for the study.

To be included in the study, the participants had to be (a) ≥18 years, (b) diagnosed with diabetes mellitus for at least 6 months prior to recruitment, (c) currently on at least one oral hypoglycaemic agent or on insulin, and (d) able to read and understand Sinhala language. Patients who were unable to read and/or understand Sinhala and those who were admitted to the hospital during the past week (since BMQ has items referring to medications used in the past week) were excluded.

### 2.2. Study Instrument and Definitions

The Brief Medication Questionnaire (BMQ) which was translated into Sinhalese as described below was used as the study instrument. The self-reported scale consists of three different screens, a 5-item regimen screen, a 2-item belief screen, and a 2-item recall screen. The overall adherence that was derived was classified as “adherent” (no positive response in all three screens), “probable adherence” (positive response in 1 screen), “probable low adherence” (positive response in 2 screens), and “low adherence” (positive response in 3 screens). In addition, we also collected the sociodemographic data of study participants, including age, gender, ethnicity, level of education, occupation, and monthly income. Furthermore, in order to evaluate the relationship between the level of adherence and glycaemic control, we evaluated the glycosylated haemoglobin (HbA1c) of the participants. Monthly income was grouped into three categories: (a) <LKR 10,000 (~US$ 65), (b) LKR 10,000–50,000 (~US$ 65–325), and (c) >LKR 50,000 (~US$ 325). Based on the Household Income and Expenditure Survey of the Department of Census and Statistics, Sri Lanka, the average monthly per capita income of the poorest 20% in the country is LKR 14,843 (~US$ 96.5), while in the richest 20% it is LKR 158,072 (~US$ 1027.5) [13].

### 2.3. Translation and Cultural Adaptation

Translation and cultural adaption was carried out following steps recommended by the WHO for the translation and adaptation of study instruments [14]. This five-step process includes (a) forward translation, (b) review of translation by experts, (c) back translation, (d) pretesting, and (e) producing the final version. The initial forward translation, from English to Sinhala was done by an independent translator, whose mother language was Sinhalese, who is familiar with terminology of the area covered by the instrument. The translator was instructed to aim for the conceptual equivalent of words/phrases, and not a word-for-word translation.

Subsequently, in the second stage a bilingual expert panel convened by the principal investigator (PR) identified and resolved the inadequate expressions/concepts of the translation. The panel included the original translator, experts in pharmacology (PG) and diabetes (PK and GRC), and those with experience in instrument development and translation (PR). The panel also modified the individual questions in order to achieve a cultural adaptation of the questionnaire. In the third stage using the same approach as that outlined in the first step, the instrument was translated back to English by an independent second translator, who has no knowledge of the original BMQ questionnaire. Discrepancies in the back translation were discussed with the expert panel, and further work was carried out until a satisfactory Sinhalese experimental version of the BMQ questionnaire was produced.

The translated experimental Sinhalese BMQ questionnaire in stage three was pretested in a sample of 10 patients with diabetes. This subset of patients was recruited from a different medical clinic other than from where patients were recruited for validation. The sample represented both males and females from different socioeconomic groups. After filling the questionnaire, each respondent was individually interviewed, where the respondents were asked what they thought the questions were asking, whether they could repeat the questions in their own words and what came to their mind when they heard a particular phrase or term. Respondents were also asked about any word they did not understand as well as any word or expression that they found unacceptable. A written report of the pretesting exercise, together with selected information regarding the participating individuals, was provided to the expert panel. The final Sinhala version of the BMQ was produced after the completion of all the steps described above. This version was used during data collection for the validation study (Annexure 1).

### 2.4. Data Collection and Biochemical Analysis

Data were collected during a period of 8 months in the medical clinics of the University Medical Unit at the National Hospital of Sri Lanka. The questionnaire on sociodemographic data (age, gender, ethnicity, level of education, and monthly income) and illness-related data was filled by the investigator and then the Sinhalese version of the self-reported BMQ was given to the recruited patients for self-completion. A blood sample (2-3 ml) was taken by a trained research assistant to measure the FBG and HbA1c. Patients were asked to attend the clinic with 8–10 hours of fasting to obtain the blood sample. Biochemical tests were performed in the laboratories of the National Diabetes Center, Colombo, Sri Lanka. HbA1c was measured by HPLC methods using a Bio-Rad D-10 analyzer (Bio-Rad, CA, USA).

### 2.5. Statistical Analysis and Validation

The translated questionnaire validation included evaluation of internal consistency, temporal stability, and performance in regards to the gold standards. It was assumed that content validity was performed by the authors of the original study. In the analysis of internal consistency, the correlation of each item with the sum of the items and interitem correlation were calculated, calculating a Cronbach *α* coefficient for each questionnaire. For the analysis of temporal stability, 30 patients with stable therapeutic schemes were retested at an interval of 30 days. Concordance between test and retest was evaluated by a gamma correlation coefficient. The performance analysis for the BMQ used the descriptive statistics of sensitivity, specificity, and area under the ROC curve, considering HbA1c as the gold standard of glycaemic control (≥8.5 as poor glycaemic control). Characteristics of the study population are also described according to the level of adherence identified by the BMQ. For the comparisons, chi-square tests, *t*-tests and Mann–Whitney tests were used according to the distribution of variables. SPSS version 14.0 was used in the analysis of data. In all analyses a *p* < 0.05 will be considered as statistically significant.

## 3. Results

### 3.1. Sociodemographic and Disease Characteristics

The total number of subjects recruited for the study was 165. The mean age (±SD) of the subjects was 60.6 ± 11.1 years (range 28–79 years), and 46.1% (*n* = 76) were males. Majority of the study participants were Sinhalese in ethnicity (64.2%, *n* = 106), educated up to GCE ordinary level (*n* = 77, 46.7%), and had a monthly income between LKR 10,000 and 50,000 (*n* = 119, 72.1%). Majority of the study population were unemployed/retired at the time of the study (58.2%, *n* = 96). The mean duration of diabetes in the study population was 13.4 ± 7.8 years (range 6 months–33 years). Mean HbA1c was 8.3 ± 1.7%, with poor glycaemic control (HbA1c ≥ 8.5%) identified in 41.8% (*n* = 69) of the patients (Table 1). Majority of the patients were on 2 drugs for the control of diabetes (55.2%, *n* = 91), and the most common drug used was metformin (81.8%, *n* = 135), followed by gliclazide (38.2%, *n* = 63), and insulin (33.9%, *n* = 56). Sociodemographic and disease characteristics are summarized in Table 1. Medication adherence measured by the BMQ regimen, belief, and recall screens were 39.4%, 75.8%, and 18.8%, respectively (Table 1).

### 3.2. Validation of Translated Questionnaire

In the analysis of temporal stability after 1 month in the subsample of 30 patients with diabetes who were on stable medication regimen, the overall BMQ and the regimen, belief, and recall screens demonstrated good concordance between test and retest with significant gamma correlation coefficients of *r* = 0.85 (*p* < 0.001), *r* = 0.81 (*p* < 0.001), *r* = 0.84 (*p* < 0.001), and *r* = 0.91 (*p* < 0.001), respectively. Analysis of the internal consistency of the BMQ was performed in the 165 patients recruited for the study. The overall BMQ (considering all three screens) had a Cronbach *α* coefficient of 0.65 (95% CI: 0.61–0.70). The Cronbach *α* coefficient of the regimen, belief, and recall screens were 0.71 (95% CI: 0.67–0.75), 0.84 (95% CI: 0.80–0.88), and 0.76 (95% CI: 0.70–0.81), respectively.

**Figure 1:**
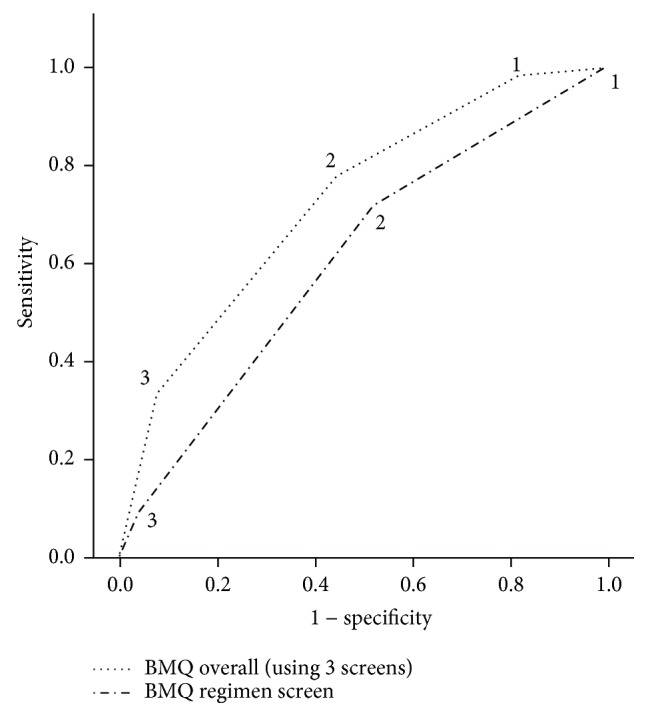
Performance of the BMQ according to the gold standard. *Note*. Brief Medication Questionnaire (overall/regimen screen): 0—adherent (no positive response); 1—probable adherence (positive response in 1 screen/one positive response); 2—probable low adherence (positive response in 2 screens/two positive responses); 3—low adherence (positive response in 3 screens/>3 positive responses).

The questionnaire performance with regards to the gold standards (poor glycaemic control—HbA1c ≥ 8.5%) is shown in Figure 1. The overall BMQ AUC was 0.73 (95% CI 0.65–0.80), while the BMQ regimen screen AUC was 0.61 (95% CI 0.53–0.70). As the number of positive responses to the questionnaires increased, the specificity in screening for low adherence also increased in relation to the gold standard (Table 2). The overall BMQ score with a cutoff value of 2 for the score of problems identified by the BMQ presented better equilibrium between sensitivity and specificity for the gold standard. This cutoff value can be utilized in screening for low adherence.

### 3.3. Association between Adherence, Sociodemographic, and Disease Factors

To study the relationship between adherence and sociodemographic and disease factors, we considered good adherence as a positive response in only 1 screen or negative responses to all questions in the 3 screens in the overall BMQ score. Low adherence was considered as the positive responses in two or more screens in the overall BMQ score. Among sociodemographic characteristics, male gender was associated with good adherence, while age, education level, or monthly income was not significantly different between the two groups (Table 3). Being only on one antidiabetic medication was associated with good adherence, while insulin therapy was associated with low adherence. Those with low adherence had a significantly higher percentage of poor glycaemic control (HbA1c ≥ 8.5%) (Table 3).

## 4. Discussion

The present study aimed at translating and validating the Sinhalese version of the Brief Medication Questionnaire (BMQ) for use in evaluating medication adherence in patients with diabetes. The translation of the original BMQ to Sinhalese and validation were done following accepted standards [14, 15]. The translated questionnaire demonstrated good reliability, temporal stability, and validity. Reliability was evaluated by the analysis of internal consistency, and the Cronbach *α* coefficient was 0.65 for the overall BMQ, with regimen, belief, and recall screens having Cronbach *α* coefficient values of 0.71, 0.84, and 0.76, respectively. Previous studies evaluating translated versions of the BMQ has demonstrated similar values for internal consistency, with the Portuguese version having a Cronbach *α* coefficient of 0.66 [8]. In a reliability analysis of a questionnaire, it is ideal when the Cronbach *α* coefficient is greater than 0.7, but values > 0.6 are considered acceptable [16]. The analysis of the BMQ showed that the regimen, belief, and recall screens performed better than the overall BMQ, with higher Cronbach *α* coefficient values.

The temporal stability (test-retest reliability) of the translated Sinhalese version of the BMQ was also evaluated. The gamma coefficients were >0.8 for overall BMQ and regimen, belief, and recall screens, demonstrating a high degree of correlation between test-retest values. Similar results for temporal stability have been observed in previous studies validating translated versions of the BMQ [8]. The performance of the Sinhalese translation of the BMQ was evaluated using HbA1c as the gold standard for glycaemic control (criterion validity). The AUC for the overall BMQ and regimen screen were 0.73 and 0.61, respectively. The overall BMQ score with a cutoff value of 2 presented better equilibrium between sensitivity (78.3%) and specificity (55.2%) and this cutoff value can be utilized in screening for low adherence. We were unable to identify previous studies evaluating BMQ in relation to control of blood glucose. In the present study, the BMQ regimen screen presented lower performance than in the original study (sensitivity of 80% versus 72.5% and specificity of 100% versus 47.9%) for the gold standard [9]. This may be due to differences in the sample, culture, and the gold standard used. However, the sensitivity and specificity of the BMQ regimen screen of a Portuguese translation (77% and 58.3) was similar to what was observed in the present study [8]. We also observed that low adherence as measured by the translated Sinhalese version of the BMQ was associated with poor glycaemic control (HbA1c > 8.5%), which provides further evidence for the validity of the translated questionnaire.

The results of the present study show that male gender and usage of only one antidiabetic medication was associated with good adherence, while insulin therapy was associated with low adherence. Adherence to antidiabetic medications is known to be associated with different factors, including age, duration and severity of disease, level of education, and monthly income [17, 18]. However, the existing scientific literature is divided with regards to the positive and/or or negative association of most of the sociodemographic and disease characteristics with adherence. For example, in a study conducted to evaluate medication adherence in Palestinian patients with diabetes, female gender was associated with good medication adherence, whereas the opposite was observed in the present analysis [17]. Jin et al., in their systematic review observed that demographic factors such as age and gender are related to a patient's various cultural, socioeconomic, and psychological backgrounds [19]. Hence, measurement of adherence using culturally validated measurement tools and identification of factors affecting low adherence in those different cultures are both equally important, in order to improve compliance and disease outcomes of a given population.

The present study has several limitations that need to be acknowledged. The lack of a practically acceptable gold standard to measure adherence was an important limiting factor. The Medication Event Monitoring System (MEMS) is currently considered the gold standard to measure adherence [20]. MEMS medication bottles contain a microelectronic chip that registers the date and time of every bottle opening. It is also known as the “imperfect gold standard,” as it could be time consuming, expensive, resource intensive, and may not be suitable for all medications/formulations [21]. In the present study, disease control as evaluated by HbA1c was used as the gold standard [8]. We also did not measure other confounding factors that could affect glycaemic control, such as dietary intake and physical activity. However, it was assumed that these factors would be evenly distributed in a large population.

## 5. Conclusions

The present study translated and validated the Sinhalese version of the Brief Medication Questionnaire, using accepted standard methods. The translated questionnaire demonstrated good reliability (internal consistency), temporal stability (test-retest reliability), and validity when assessed using a gold standard for disease control. Using culturally validated tools to evaluate adherence may help clinicians to identify low adherence and institute appropriate corrective measures to improve disease outcomes.

## Figures and Tables

**Table 1:** Sociodemographic and disease characteristics of the study population.

Variable	Number	Percentage (%)
Gender		
Male	76	46.1
Female	89	53.9
Ethnicity		
Sinhalese	106	64.2
Muslim	43	26.1
Tamil	15	9.1
Other	1	0.6
Level of education		
No formal education	7	4.2
Grades 1–5	11	6.7
Grades 6–10	40	24.2
Up to GCE O/L	77	46.7
Up to GCE A/L	19	11.5
Higher education	11	6.7
Monthly income		
<LKR 10,000 (~US$ 65)	41	24.8
LKR 10,000–50,000 (~US$ 65–325)	119	72.1
>LKR 50,000 (~US$ 325)	5	3.0
Occupation		
Retired/unemployed	96	58.2
Manual worker	27	16.4
Managerial worker	16	9.7
Clerical worker	6	3.6
Other	20	12.1
Comorbid diseases		
Hypertension	95	57.6
Hyperlipidaemia	64	38.8
Ischaemic heart disease	47	28.5
Bronchial asthma	15	9.1
Number of antidiabetic medications		
One	58	35.2
Two	91	55.2
Three	16	9.7
Type of antidiabetic medication		
Metformin	135	81.8
Gliclazide	63	38.2
Insulin	56	33.9
Sitagliptin	9	5.4
Glibenclamide	7	4.2
Poor glycaemic control (HbA1c ≥ 8.5%)	69	41.8
Medication adherence (BMQ)		
Regimen screen (no positive response)	65	39.4
Belief screen (no positive response)	125	75.8
Recall screen (no positive response)	31	18.8
Overall adherence		
Adherent (no positive response)	18	10.9
Probable adherence (positive response in 1 screen)	47	28.5
Probable low adherence (positive response in 2 screens)	46	16.4
Low adherence (positive response in 3 screens)	54	32.8

**Table 2:** Performance of the BMQ according in comparison to gold standard.

	Sensitivity (%)	Specificity (%)
Brief Medication Questionnaire (overall)		
Adherent (no positive response)	100	0
Probable adherence (positive response in 1 screen)	98.6	17.7
Probable low adherence (positive response in 2 screens)	78.3	55.2
Low adherence (positive response in 3 screens)	33.3	92.7
BMQ regimen screen		
Adherent (no positive response)	100	0
Probable adherence (one positive response)	72.5	47.9
Probable low adherence (two positive responses)	31.9	79.2
Low adherence (≥3 positive responses)	1.4	100

**Table 3:** Relationship between adherence, sociodemographic, and disease characteristics.

Variable	Number (%)		*p* value
	Good adherence	Low adherence	
Sociodemographic characteristics			
Age (years)	60.4 ± 12.8^∗^	61.0 ± 9.7^∗^	0.73
Male gender	38 (55.9)	38 (39.2)	0.04
Education GCE O/L and above	46 (67.6)	61 (62.9)	0.62
Monthly income > LKR 10,000	54 (79.4)	70 (72.2)	0.36
Comorbid diseases			
Hypertension	45 (66.2)	50 (51.5)	0.07
Hyperlipidaemia	26 (38.2)	38 (39.2)	0.52
Ischaemic heart disease	13 (19.1)	34 (35.1)	0.03
Bronchial asthma	4 (5.9)	11 (11.3)	0.28
Type of antidiabetic medication			
Metformin	57 (83.8)	77 (79.4)	0.55
Sulphonylurea	36 (52.9)	42 (43.3)	0.27
Insulin	10 (14.7)	42 (43.3)	<0.001
Poor glycaemic control (HbA1c ≥ 8.5%)	15 (22.1)	54 (55.7)	<0.001

^∗^Mean ± SD.

## Data Availability

The datasets used and/or analyzed during the current study are available from the corresponding author on reasonable request.

## Supplementary Materials

Supplementary Materials Annexure 1: Sinhalese translation of the Brief Medication Questionnaire used for validation.
