# Supplementary material for: Translation and Validation of the Sinhalese Version of the Brief Medication Questionnaire in Patients with Diabetes Mellitus
Source: J Diabetes Res. 2018 May 23;2018:7519462. doi: 10.1155/2018/7519462 (PMC5989170; doi:10.1155/2018/7519462)
Supplement: Supplementary Materials — Annexure 1: Sinhalese translation of the Brief Medication Questionnaire used for validation. [file 7519462.f1.pdf]

<u>දියවැඩියා ඖෂධ භාවිතය පිළිබඳ **කෙටි** ප්‍රශ්නාවලිය</u>

(Brief Medication Questionnaire on Diabetes – BMQ)

මෙම ප්‍රශ්නාවලිය ඔබ දැනට <u>**දියවැඩියාව**</u> සඳහා ගන්නා ඖෂධ ගැන <u>**පමණක්**</u> අසනු ලබයි.

ඔබ <u>**දියවැඩියාව**</u> සඳහා ගනිමින් සිටින සෑම ඖෂධයක්ම මෙහි කරුණාකර සඳහන් කරන්න.

1. ඔබ අද දින ඔබේ <u>**දියවැඩියා ඖෂධ**</u> ඔබ සමඟ සායනයට රැගෙනවිත් තිබේද?

    1. නැත   2. ඔව්

2. ඔබ <u>**දැනට**</u> **දියවැඩියාව** සඳහා කොපමණ ඖෂධ වර්ග ගණනක් භාවිතා කරන්නේද?

    ඖෂධ ගණන ………….

3. ඔබ <u>**දැනට**</u> **දියවැඩියාව** සඳහා ගන්නා ඖෂධ මොනවාද?

    <u>**ඖෂධ නාමය හෝ ඖෂධ ගැන විස්තරයක්** (උදා :ඖෂධ මාත්‍රාව, දිනකට ගන්නා වාර ගණන</u>)

    ඖෂධ **A**: ………………………………………………..

    ඖෂධ **B**: ………………………………………………..

    ඖෂධ **C**: ………………………………………………..

    ඖෂධ **D**:………………………………………………..

4. ඔබ <u>**පසුගිය මාස 6 තුළ**</u> **දියවැඩියාව** සඳහා භාවිතා කල කිසියම් ඖෂධයක් නතර කළාද?
    ඔව්/නැත

    එසේ නතර කළා නම් ;

    a. නතර කළ ඖෂධය   b. නතර කළ හේතුව

    1. …………………………………   ……………………………
    2. …………………………………   ……………………………

පහත සඳහන් ප්‍රශ්න **<u>පසුගිය සතියේ</u>** ඔබේ දියවැඩියා ඖෂධ භාවිතය පිළිබඳවයි.

කරුණාකර එක් එක් ප්‍ර…ශ්න සඳහා, ඔබේ අත්දැකීම් හොඳම ආකාරයෙන් විස්තර කරන **අංකය/අංක** ලකුණු කරන්න. ඔබ **<u>ඉහත</u>** ලැයිස්තුගත කල එක් එක් ඖෂධය සඳහා (**A, B, C,D**) වෙන වෙනම ප්‍රශ්නවලට පිළිතුරු සපයන්න.

ඖෂධ **A**:……………………………………

5 මෙම ඖෂධය කොපමණ වතාවක් භාවිතා කිරීමට වෛද්‍යවරයා උපදෙස් ලබාදී තිබේද?

1. සෑම දිනකම 2. අවශ්‍ය විටදී පමණක් 3. නොදනිමි

6. මෙම ඖෂධය ඔබට උපකාරවන **ආකාරය/ආකාර** වන්නේ; (පිළිතුරු කිහිපයක් ලකුණු කල හැක)

1. මගේ සීනි මට්ටම වැඩි කිරීමෙන්
2. මගේ සීනි මට්ටම අඩු කිරීමෙන්
3. බර අඩු කිරීමෙන්
4. හෘද රෝග වැළැක්වීමෙන්
5. ආහාර රුචිය අඩු කිරීමෙන්
6. වෙනත් (සඳහන් කරන්න)……………………………………………
7. නොදනිමි

7. පහත සඳහන් ප්‍රශ්න **<u>පසු ගිය සතිය තුල</u>** ඔබගේ දියවැඩියා ඖෂධ භාවිතය පිළිබඳවයි;

a. ඔබ පසුගිය සතිය තුල මෙම ඖෂධය යම් ප්‍රමාණයක් හෝ භාවිතා **කළාද**?

1. නැත 2. ඔව්

b. ඔබ පසු ගිය සතිය තුල **<u>දින කීයක්</u>** මෙම ඖෂධය භාවිතා **කළාද**?

මම මෙම ඖෂධය දින: 0 1 2 3 4 5 6 7 ක් භාවිතා කළෙමි.

c. පසුගිය සතිය තුල ඔබ සාමාන්‍යයෙන් මෙම ඖෂධය **<u>දිනකට කොපමණ වරක්</u>** භාවිතා කළාද?

මම මෙම ඖෂධය දිනකට: 0 1 2 3 වරක් භාවිතා කළෙමි.

d. පසු ගිය සතිය තුල ඔබ සාමාන්‍යයෙන් **<u>එක් වරකදී</u>** මෙම ඖෂධය කොපමණ ප්‍රමාණයක් භාවිතා කළාද?

මම මෙම ඖෂධය **<u>එක් වරකදී</u>** පෙති/කරල්: 0 1 2 3 ක් භාවිතා කළෙමි

e. පසු ගිය සතිය තුල ඔබට මෙම ඖෂධය ගැනීමට කොපමණ වරක් **<u>නොහැකි</u>** වුණාද?

මා හට මෙම ඖෂධය ගැනීමට: 0 1 2 3 4 5 6 7 වරක් නොහැකි වුනි.

8. ඔබට කොතරම් හොඳින් මෙම ඖෂධය ක්‍රියා කරන්නේ ද?

1. කිසිසේත්ම ක්‍රියා නොකරයි
2. මධ්‍යස්ථ වශයෙන් ක්‍රියා කරයි
3. ඉතා හොඳින් ක්‍රියා කරයි
4. නොදනිමි

9. ඔබට මෙම ඖෂධය නිසා අපහසුතාවයන් ඇතිවේද?

1. කිසිවිටක නැත
2. තරමක් අපහසුතා ඇතිවේ
3. බොහෝ අපහසුතා ඇතිවේ
4. නොදනිමි

10. මෙම ඖෂධය භාවිතා කරන විට ඔබට පහත අපහසුතා කොපමණ ප්‍රමාණයකට ඇතිවේද ?

	කිසිවිටක නැත	මද වශයෙන්	බොහෝ වීට
a. සියලු මාත්‍රා නියමිත කාලයට ගැනීම අපහසුය	0	1	2
b. මෙම ඖෂධය සඳහා වියදම දැරීමට අපහසුය	0	1	2
c. ඖෂධ අවසන්වූ පසු නැවතත් නියමිත කාලයට ලබා ගැනීම	0	1	2
d. මෙම ඖෂධය නිසා මා හට තවමත් අනවශ්‍යය අතුරු ආබාධ ඇතිවේ	0	1	2
e. මම මෙම ඖෂධයේ දිගු කාලීන බලපෑම් ගැන වද වෙමි	0	1	2
f. මෙම ඖෂධය පිළිබඳ මා හට වෙනත් ගැටලු හෝ ප්‍රශ්න ඇත	0	1	2
